# Which is the better choice? A clinical cohort study protocol evaluating the differences between standard medial parapatellar and minimally invasive approaches in total knee replacement

**DOI:** 10.1097/MD.0000000000024209

**Published:** 2021-01-08

**Authors:** Qiong Jia, Xin Chen, Jun Zhang, Yang Hu

**Affiliations:** a The First Clinical Medical College, Guangzhou University of Chinese Medicine, Guangdong; b Department of Surgical Nursing, Hunan Traditional Chinese Medical College; c Department of Orthopedics, The First Affiliated Hospital of Hunan College of traditional Chinese Medicine, Hunan, China.

**Keywords:** medial parapatellar approach, minimally invasive approach, protocol, retrospective, total knee replacement

## Abstract

**Background:**

There is still a lack of consensus on the best approach for total knee replacement (TKR). We carried out this present retrospective cohort study to assess the overall safety and effectiveness of a minimally invasive approach without the use of computer navigation in comparison with conventional TKR.

**Methods:**

A retrospective review of patients who receiving the primary TKR in the same institution from 2014 to 2016 was conducted. The inclusion criteria for the study indicated that the patient required a unilateral or bilateral TKR was between 18 and 80 years’ old, provided informed consent, was followed up for at least 2 years, and was in stable health. There was no treatment for any condition or condition that might pose a risk of excessive surgery. The same TKR standard rehabilitation program was provided to all patients. Data were collected on patient demographics, anesthesia style, American Society of Anesthesiology scores, tourniquet duration, and surgical drainage loss. Our primary outcome measure was discharge time. Secondary outcomes included duration of surgery, incidence of postoperative complications, imaging location 6 weeks after surgery, Oxford Knee Score, Western Ontario and McMaster Universities Arthritis Index, and knee ROM. Complications were recorded and classified as surgical site, thromboembolic, systemic, or requiring reoperation.

**Results:**

It was assumed that there is a remarkable difference in postoperative outcomes between the 2 groups.

**Conclusion:**

The limitations of our present research include the inherent limitations in any retrospective cohort research, including the observation bias and possibility of selection.

**Trial registration:**

This study protocol was registered in Research Registry (researchregistry6349).

## 1 Introduction

In the United States, nearly 700,000 total knee replacements (TKRs) are performed annually to reduce the pain and disability associated with knee osteoarthritis. Future projections suggest that by the year 2030, 3.48 million TKRs will be performed annually.^[1–3]^ The most common exposure for TKR is the medial parapatellar approach using a standard incision. Despite the satisfying surgical outcomes, this approach has been criticized because it causes extensive damage to the extensor mechanisms of the knee and can adversely affect the patellar blood supply.^[4,5]^

Over the past decade, several authors have demonstrated that minimally invasive surgical TKA techniques can be performed reliably and that they are acceptable in routine clinical practice. The primary objectives of the minimally invasive surgical TKR are to minimize surgical trauma, reduce postoperative pain, and improve postoperative rehabilitation and early functional recovery.^[6–8]^ However, other authors also raised concerns about the possibility of perioperative complications, component malposition issues, and the lack of compelling data on long-term benefits from minimally invasive surgical techniques.^[9,10]^ Although a large number of published studies have compared the minimally invasive approach with the standard medial parapatellar approach on the medial side, there is still a lack of consensus on the best method for TKR.^[11–14]^

Therefore, we carried out this present retrospective cohort study to assess the overall safety and effectiveness of a minimally invasive approach without the use of computer navigation in comparison with conventional TKR. It was assumed that there is a remarkable difference in postoperative outcomes between the 2 groups.

## 2 Materials and methods

### 2.1 Patients

The inclusion criteria for the study indicated that the patient required a unilateral or bilateral TKR, was between 18 and 80 years’ old, provided informed consent, was followed up for at least 2 years, and was in stable health. There was no treatment for any condition or condition that might pose a risk of excessive surgery. Patients were excluded if they were known to have insufficient femoral or tibial bone stock, a body mass index of >35 kg/m^2^, a failed total or unicondylar knee replacement of the affected knee, an active local or systemic infection, collateral ligament insufficiency, knee flexion of <90°, a fixed flexion deformity of >15°, a varus or valgus deformity of >20°, and/or an immunosuppressive disorder, such as acquired immunodeficiency syndrome.

### 2.2 Study design

A retrospective review of patients who receiving the primary TKR in the same institution from 2014 to 2016 was conducted. This current retrospective cohort study was approved through Institutional Review Board in the First Affiliated Hospital of Hunan College of traditional Chinese Medicine (HN20200813) and was registered in the research registry (researchregistry6349). If patients met these inclusion and exclusion criteria, they would be divided into 1 of 2 treatment groups: minimally invasive approach group and standard medial parapatellar approach group.

### 2.3 Surgery technique

For patients in the minimally invasive approach group, a mini-midvastus approach was used, and the patella was not everted, whereas for patients in the standard medial parapatellar approach group, a medial parapatellar approach was used, and the patella was everted. In both groups, the flexion and extension spaces were equalized, and soft tissues were balanced. For all patients, a tourniquet was used, and the posterior cruciate ligament was removed; the patella was resurfaced. All the components were fixed with cements. The joint capsule and retinaculum were sutured with the knee position at a flexion angle of 90 degree. A Scorpio NRG PS Total Knee System (Stryker Orthopedics, Mahwah, NJ) was used for all the knees.

### 2.4 Postoperative management

The same TKR standard rehabilitation program was provided to all patients. Quadriceps strengthening exercises and range of motion and weight-bearing walking were performed from the day after surgery. Active range of motion (ROM) was encouraged and full weight-bearing ambulation was allowed. Discharge was allowed when they were able to ambulate 30 m, ascend and descend 3 steps, and had pain well controlled with oral medications. Patients were sent home for 3 specific knee ROM exercises and were encouraged to seek formal physical therapy on an outpatient basis twice or 3 times a week for the first month.

### 2.5 Outcomes

Data were collected on patient demographics, anesthesia style, American Society of Anesthesiology scores, tourniquet duration, and surgical drainage loss. Our primary outcome measure was discharge time. This was measured as the time from surgery until the time patients were documented to be ready for discharge by the surgical and physiotherapy teams. Secondary outcomes included duration of surgery, incidence of postoperative complications, imaging location 6 weeks after surgery, Oxford Knee Score, Western Ontario and McMaster Universities Arthritis Index, and knee ROM. Complications were recorded and classified as surgical site, thromboembolic, systemic, or requiring reoperation.

### 2.6 Statistical analysis

Intention-to-treat analysis was used for all clinical outcome variables and was performed by an independent blinded external statistician. The Student *t* test was used to determine any differences between intraoperative variables and for univariate comparison of postoperative parameters. Treatment comparisons for the continuous postoperative outcome variables were based on a marginal linear model, with the preoperative level of a variable used as a part of the outcome vector. Inferences on the correlation structure were based on a likelihood ratio test. On the basis of these, an unrestricted correlation structure was assumed in all models. Linear contrasts of fitted model estimates were constructed and used to test the hypotheses of interest. Two-tailed tests were used throughout. Two-sided *P* values of <.05 were considered to indicate significance.

## 3 Discussion

TKR is routinely performed through the medial parapatellar approach, which provides excellent exposure and reproducible long-term effects. However, this approach requires femoral quadriceps tendon division, patella eversion, and anterior tibial subluxation, which may exacerbate postoperative pain and hinder recovery.^[2]^ The technique of preserving the minimally invasive quadriceps femoris is designed to improve postoperative function and reduce pain. Functional benefits have been demonstrated but at the potential expense of component position and concerns regarding early revision.^[5,8]^ Therefore, we carried out this present retrospective cohort study to assess the overall safety and effectiveness of a minimally invasive approach without the use of computer navigation in comparison with conventional knee arthroplasty. It was assumed that there is a remarkable difference in postoperative outcomes between the 2 groups. The limitations of our present research include the inherent limitations in any retrospective cohort research, including the observation bias and possibility of selection.

## Author contributions

QJ and XC conceived, designed, and planed the study. QJ, JZ, and XC are recruiting the study participants and performing the interventions. YH supervised the study. QJ, JZ, and XC will interpret and analyze the data. QJ drafted the manuscript. XC and YH critically revised the manuscript for important intellectual content. All authors have full access to the manuscript and take responsibility for the study design. All authors have approved the manuscript and agree with submission.

**Data curation:** Qiong Jia, Xin Chen.

**Formal analysis:** Qiong Jia, Xin Chen.

**Funding acquisition:** Yang Hu.

**Investigation:** Qiong Jia, Xin Chen.

**Methodology:** Xin Chen.

**Project administration:** Yang Hu.

**Resources:** Yang Hu.

**Software:** Jun Zhang.

**Validation:** Jun Zhang.

**Visualization:** Jun Zhang.

**Writing – original draft:** Qiong Jia.

**Writing – review & editing:** Jun Zhang, Yang Hu.
